# Fluid and White Matter Suppression Imaging and Voxel-Based Morphometric Analysis in Conventional Magnetic Resonance Imaging-Negative Epilepsy

**DOI:** 10.3389/fneur.2021.651592

**Published:** 2021-04-29

**Authors:** Ke Sun, Tao Yu, Dongju Yang, Zhiwei Ren, Liang Qiao, Duanyu Ni, Xueyuan Wang, Yongxiang Zhao, Xin Chen, Jing Xiang, Nan Chen, Runshi Gao, Kun Yang, Yicong Lin, Tobias Kober, Guojun Zhang

**Affiliations:** ^1^Beijing Institute of Functional Neurosurgery, Xuanwu Hospital, Capital Medical University, Beijing, China; ^2^Department of Neurology, Xuanwu Hospital, Capital Medical University, Beijing, China; ^3^Department of Radiology, Xuanwu Hospital, Capital Medical University, Beijing, China; ^4^Department of Neurology, MEG Center, Cincinnati Children's Hospital Medical Center, Cincinnati, OH, United States; ^5^Department of Evidence-Based Medicine, Xuanwu Hospital, Capital Medical University, Beijing, China; ^6^Advanced Clinical Imaging Technology, Siemens Healthcare, Lausanne, Switzerland; ^7^Department of Radiology, Lausanne University Hospital and University of Lausanne, Lausanne, Switzerland; ^8^LTS5, École Polytechnique Fédérale de Lausanne, Lausanne, Switzerland

**Keywords:** epilepsy, non-lesional, flaws, voxel-based morphometric, MRI post-processing, focal cortical dysplasia

## Abstract

**Purpose:** Delineation of subtle lesions in magnetic resonance imaging (MRI)-negative patients is of great importance in preoperative epilepsy evaluation. The aim of our study was to explore the diagnostic value of the novel fluid and white matter suppression (FLAWS) sequence in comparison with a voxel-based MRI postprocessing morphometric analysis program (MAP) in a consecutive cohort of non-lesional patients.

**Methods:** Surgical candidates with a negative finding on an official neuroradiology report were enrolled. High-resolution FLAWS image and MAP maps generated based on high-resolution three-dimensional (3D) T1 image were visually inspected for each patient. The findings of FLAWS or MAP-positive (FLAWS/MAP+) regions were compared with the surgical resection cavity in correlation with surgical outcome and pathology.

**Results:** Forty-five patients were enrolled; the pathological examination revealed focal cortical dysplasia (FCD) in 32 patients and other findings in 13 patients. The positive rate, sensitivity, and specificity were 48.9%, 0.43, and 0.87, respectively, for FLAWS and 64.4%, 0.57, and 0.8, respectively, for MAP. Concordance between surgical resection and FLAWS+ or MAP+ regions was significantly associated with a seizure-free outcome (FLAWS: *p* = 0.002; MAP: *p* = 0.0003). A positive finding in FLAWS and MAP together with abnormalities in the same gyrus (FLAWS–MAP gyral+) was detected in 31.1% of patients. FLAWS+ only and MAP+ only were found in 7 (15.5%) and 14 (31.1%) patients, respectively.

**Conclusions:** FLAWS showed a promising value for identifying subtle epileptogenic lesions and can be used as a complement to current MAP in patients with MRI-negative epilepsy.

## Introduction

Thirty percent of patients with epilepsy are pharmacoresistant (1). Surgical resection of the epileptogenic zone has been shown to be an effective treatment option (2). However, good surgical outcome depends on accurate delineation and complete resection of the epileptogenic zone (3). Approximately 20–40% of surgical candidates have negative results on conventional magnetic resonance imaging (MRI) (4). The absence of a lesion on MRI may lead to insufficient evidence for forming a surgical hypothesis and is associated with poor surgical outcome (4). In order to identify epileptic abnormalities with a higher efficiency and guide the implantation of intracranial electrodes in MRI-negative epilepsy patients, novel imaging acquisition techniques and advanced imaging analysis algorithms are needed.

The fluid and white matter suppression (FLAWS) sequence, a recently developed MRI sequence, was first proposed by Tanner et al. (5) and derived from the magnetization-prepared two rapid acquisition gradient-echoes (MP2RAGE) sequence. In FLAWS, the two inversion contrasts of the MP2RAGE are configured so that the first contrast determined by the inversion time (TI1) produces a white matter (WM)-nulled image, while the second, acquired at TI2, generates a cerebrospinal fluid (CSF)-suppressed image. Therefore, the minimum voxel-wise values of the two aforementioned images generate a gray matter (GM)-specific FLAWS contrast image. Owing to this advantage, previous studies have used this sequence for fast brain tissue segmentation (6) and subcortical nucleus separation (7). However, reports of FLAWS in epilepsy are rare because of its novelty. In a pilot study, we found that FLAWS was superior to conventional MRI sequences [T1, T2, and fluid-attenuated inversion recovery (FLAIR)] in visually identifying epileptic lesions among patients with both MRI-positive and MRI-negative focal cortical dysplasia (FCD) (8).

In addition to novel imaging acquisition protocols, many postprocessing methods using mature MRI images have been well-developed and utilized in clinical work. These strategies include curvilinear reformatting (9, 10), texture analysis of T1-weighted volumetric MRI (11), and quantitative voxel-based intensity analysis of T2 images (12) or FLAIR images (13). Among these strategies, a voxel-based MRI morphometric analysis program (MAP) implemented in the statistical parametric mapping software (SPM), which can improve the visualization and detection of subtle lesions with high sensitivity (14, 15), was proposed and validated in epilepsy (16, 17).

The aim of this study was 2-fold. Since the detection of subtle abnormalities in conventional MRI-negative epilepsy is both challenging and worth exploring for presurgical evaluation, based on our previous findings, we decided to focus only on MRI-negative patients. In order to improve the study representativeness, the design aimed to consecutively recruit surgical candidates from our epilepsy center. Thus, the first aim was to evaluate the potential value of FLAWS in detecting epileptic lesions in patients with MRI-negative epilepsy. FLAWS is relatively new and has not yet been routinely used in clinical practice. Conversely, MAP is a valuable, well-developed, and practical tool and widely used in >30 centers (18). Hence, the second aim was to test the practical value of FLAWS through comparison of the FLAWS yield with that by MAP. We designed the study to include non-lesional surgical candidates to acquire FLAWS images and conduct MAP analysis. FLAWS and MAP findings were compared to surgical resection in correlation with surgical outcome. We hypothesized that FLAWS alone would reveal subtle lesions in clinical cohorts and that a complementary value exists in FLAWS with MAP.

## Methods

### Patient Recruitment and Procedure

Patients with non-lesional pharmacoresistant epilepsy referred for presurgical evaluation at the Comprehensive Epilepsy Center of Beijing, Xuanwu Hospital, from January 2017 to August 2018, were consecutively included for FLAWS image scanning. The inclusion criteria were as follows: (1) a negative finding on the official neuroradiology report before the multidisciplinary presurgical evaluation conference (MPEC), (2) MPEC recommended resective surgery, (3) a T1-weighted magnetization-prepared rapid acquisition with gradient-echo (MPRAGE) image was available for MAP analysis, and (4) consent for FLAWS scan and participation in this study. The exclusion criteria were (1) age <5 years old at screening, (2) FLAWS was not conducted because of organizational problems, (3) resective epilepsy surgery was not performed, (4) poor imaging quality of conventional MRI or FLAWS image, and (5) the patient was lost to follow-up or had <12 months of follow-up.

This study was approved by the Ethics Committee of Xuanwu Hospital and conducted in accordance with the Declaration of Helsinki. Informed written consent was obtained from each patient or legal guardian if the patient was underage.

### Presurgical Evaluations, Surgery, and Pathology

The therapeutic schedule regarding intracranial electrode implantation and the surgical strategy for all patients were discussed at the routine MPEC with neuroradiologists, electrophysiologists, neurologists, neurosurgeons, and researchers dedicated to epilepsy, at the Comprehensive Epilepsy Center of Beijing, Xuanwu Hospital, based on information from semiology, video electroencephalogram (EEG), routine MRI, positron emission tomography, and magnetoencephalography (MEG). Generally, consistent with the guidelines (19), investigation with intracranial electrode sampling is considered when patients have the following conditions: (1) no MRI lesion, (2) insufficiently congruent anatomical–electroclinical information from the non-invasive examinations, and (3) inconclusive localization of the suspected epileptogenic zone or early involvement of the eloquent cortex. The results of FLAWS and MAP were not used for clinical diagnosis and treatment recommendations during the study period. Additional details on the presurgical evaluation can be found elsewhere (20).

The clinical routine MRI protocol for epilepsy at our hospital was acquired using a 3.0-T MRI scanner system (Siemens, Erlangen, Germany), including T1WI [repetition time (TR)/echo time (TE), 160/3.05; matrix, 256 × 205; field of view (FOV), 240 mm; axial; slices thickness, 3.0 mm], T2WI (TR/TE, 160/3.05; matrix, 256 × 256; FOV, 240 mm; axial; slices thickness, 3.0 mm), 2D-FLAIR (TR/TE, 8,500/85; matrix, 256 × 180; FOV, 240 mm; axial; slices thickness, 3.0 mm), 3D-FLAIR (TR/TE, 8,500/88; matrix, 256 × 256; FOV, 230 mm; coronal; slices thickness, 3.0 mm), and DWI (TR/TE, 5,500/90; matrix, 136 × 136; FOV, 240 mm; axial; slices thickness, 3.0 mm). An MPRAGE image was acquired on patients who underwent MEG. The MRI image of each patient was re-reviewed at the MPEC. Patients initially reported as “negative” were divided into the “subtly lesional” or the “strictly non-lesional” subgroups depending on whether a subtle abnormality was found at MPEC.

An individualized, tailored resection was performed based on the conclusion from converging information by the MPEC. Tissues obtained were sent for pathological examination with experienced neuropathologists dedicated to epilepsy, following the International League Against Epilepsy classification (21, 22).

Patients were followed up by outpatient consultation and telephone interviews at 3, 6, and 12 months postoperatively and at longer intervals thereafter. The surgical outcome was evaluated, according to Engel's classification (23).

### FLAWS Acquisition

FLAWS images were acquired using a prototype sequence with a 12-channel head-neck coil in the 3.0-T MRI scanner system (Siemens, Erlangen, Germany; TR/TE, 5,000/2.88; matrix, 256 × 256; FOV, 256 mm; sagittal; slices thickness, 1.0 mm). Two TIs were used to generate the white matter-nulled images (TI1: 409 ms; FLAWS1) and cerebrospinal fluid-nulled images (TI2: 1,100 ms; FLAWS2). The FLAWS image was generated by taking the minimum voxel values of FLAWS1 and FLAWS2. Other details of the scan parameters were previously reported (8).

### MAP Maps Calculation

MAP was performed based on the MPRAGE image (Siemens, Erlangen, Germany; TR/TE, 1,900/2.2; matrix, 256 × 256; FOV, 250 mm; sagittal; slices thickness, 1.0 mm; TI, 900 ms) using the SPM12 toolbox (Wellcome Department of Cognitive Neurology, London, UK) in MATLAB 2017a (MathWorks, Natick, MA, USA) following established methods (14, 16). Three statistical maps highlighting deviations from a normal database were generated by MAP analysis: junction (sensitive to blurring of GM–WM junction), extension (sensitive to abnormal gyration and extension of GM into WM), and thickness (sensitive to abnormal cortical thickness) map (17). The main analysis included steps as normalization of the individual brain into the standard brain; brain segmentation into GM, WM, and CSF; and comparison with the normal database. Details can be found elsewhere (15, 16). The normal database, consisting of 150 subjects (70 females, 80 males; mean age: 30.9 years; range 15–77 years), was kindly provided with the MAP program and has been tested in many studies with patients' age ranging from 2 to 66 years old (16, 17, 24, 25).

### FLAWS and MAP Analysis Procedures

Since information from one study could influence the judgment of another, FLAWS and MAP maps were analyzed by different investigators in the following order (Supplementary Figure 1):

- Step 1: Independent original FLAWS visual inspection without electroclinical information, MPRAGE, MAP maps, and postoperative computed tomography (CT) image, aiming to report FLAWS-positive (FLAWS+) regions and feature scores. Two experienced radiologists (XC, NC), both with >10 years of experience, reviewed the images. Independent MAP analysis, without electroclinical information, FLAWS, and postoperative CT image, aimed to report MAP-positive (MAP+) regions in junction, extension, and thickness maps. Two experienced epileptologists (DY, XW) with >10 years of experience in epilepsy reviewed the maps.
- Step 2: Determination of the relationship between the resection cavity and FLAWS or MAP abnormalities was performed with the registration and segmentation module in 3D Slicer (website: http://www.slicer.org).
- Step 3: A joint re-review of co-registered FLAWS and MAP was conducted to determine concordance or discordance between the FLAWS+ and MAP+ regions (XC and DY).

### Independent Original FLAWS Analysis

The original FLAWS images were evaluated with regard to the detection of structural abnormalities. Five typical features have been reported as helpful for the visual identification of subtle lesions on MRI and were used in our study: (A), abnormal cortical morphology, including thinning and thickening (defined as a change in the thickness of at least half of the normal cortex) and abnormal deep sulci; (B), abnormal cortical signal intensity; (C), blurred GM–WM junction; (D), abnormal signal intensity of subcortical WM; and (E), transmantle sign (8, 26). False-positive features such as thin thread-like and inhomogeneous signals were rejected. Two neuroradiologists (XC, NC) reviewed FLAWS images to determine whether a FLAWS+ region existed and where the FLAWS+ region was located. FLAWS+ regions were scored with the five aforementioned features by comparing to the signal in the contralateral side (0, no abnormality; 1, abnormality). Discrepancies in the assessment were resolved by consensus between the two radiologists.

### Independent MAP Analysis

Based on the literature, the *z*-score thresholds of 4, 6, and 4 were used for identifying candidate MAP+ regions on the junction, extension, and thickness map, respectively (17, 27, 28). Non-specific WM change or high *z*-score regions caused by technical issues were rejected. Two epileptologists (DY, XW) visually reviewed the three maps to determine whether a MAP+ region existed, the location of the MAP+ region, and the MAP+ region score on the three maps (0, no abnormality; 1, abnormality). The abnormality was reaffirmed by checking with the original MPRAGE image to confirm the MAP+ region.

### Comparison Between Resection Cavity and Detected Abnormalities

The determination of the relationship between the resection cavity and FLAWS or MAP abnormalities was performed using registration, segmentation, and ruler measurement function in 3D Slicer (KS). First, FLAWS and postoperative CT were registered onto the preoperative MPRAGE using the 3D Slicer individually, which allowed us to analyze the images in the same coordinate system. Registration was unnecessary for MAP maps because they were generated based on MPRAGE. Second, delineation of the resection cavity on the postoperative CT with the segmentation module in 3D Slicer allowed visualization of the surgical margin on MPRAGE, co-registered FLAWS, and co-registered MAP maps, on axial, sagittal, and coronal views. Third, the co-registered FLAWS or MAP was further overlaid with the co-registered postoperative CT to assess the relationship between the resection cavity and FLAWS or MAP abnormalities. The correlation was classified as “concordance” or “nonconcordance,” depending on the distance between the outermost border of the lesion and the surgical resection margin measured using the ruler in the 3D Slicer. Considering the tissue movement around the resection cavity after surgery, “concordance” was only determined if the border was completely within or <8 mm outside of the resection margin, similar to a previous study (29). If multiple positive lesions existed, every lesion needed to meet the aforementioned criterion to be considered as “concordance.” Otherwise, the findings were regarded as “nonconcordance” (Supplementary Figure 2).

### Joint Re-Review of the Co-Registered FLAWS and MAP Maps

Co-registered FLAWS and MAP maps were overlaid on the 3D Slicer software to determine whether the FLAWS+ and MAP+ regions were concordant (XC and DY). Concordance was defined at the gyral or lobar level, depending on whether the FLAWS+ and MAP+ regions coexisted in the same anatomic gyrus or the same lobe. Discordance was defined if the FLAWS+ and MAP+ regions are located in different lobes or hemispheres.

### Statistics

Patients were divided into two groups: seizure-free group (Engel I) and not seizure-free group (Engel II–IV), based on the surgical outcome at 12 months. The Pearson chi-square test was used to estimate the correlation of FLAWS or MAP-positive regions with resection to surgical outcome and the difference in age group, gender, resection type, pathology, and invasive exploration before surgery, as well as “MRI subgroups” between the two groups; Fisher's exact test was used when the chi-square test included expected values <5. The *t* and Wilcoxon rank-sum tests were used to compare differences in age and seizure duration between the seizure-free group and the not seizure-free group.

The positive rate, sensitivity, specificity, positive predictive value (PPV), and negative predictive value (NPV) were calculated for FLAWS and MAP in the entire cohort. The positive rate was defined as the number of FLAWS/MAP+ cases/the number of the whole dataset. Pearson chi-square test was used to estimate the difference in positive rate between FLAWS and MAP in the overall cohort as well as to estimate the difference between temporal and extratemporal, “subtly lesional” and “strictly nonlesional” subgroups; Fisher's exact test was used when the chi-square test included an expected value <5. True positive was defined as concordance between the FLAWS/MAP findings and resection with good surgical outcome, false positive as concordance between the FLAWS/MAP findings and resection with poor surgical outcome, true negative as non-concordance between the FLAWS/MAP findings and resection with poor surgical outcome, and false negative as non-concordance between FLAWS/MAP findings and resection with good surgical outcome. These definitions were based on those of previous reports in the literature (18, 30).

All statistical analyses were performed using R, version 3.6.3 (R Foundation for Statistical Computing, r-project.org), with statistical significance defined as *p* < 0.05 (two-sided).

## Results

### Demographic and Clinical Data

**Figure 1:**
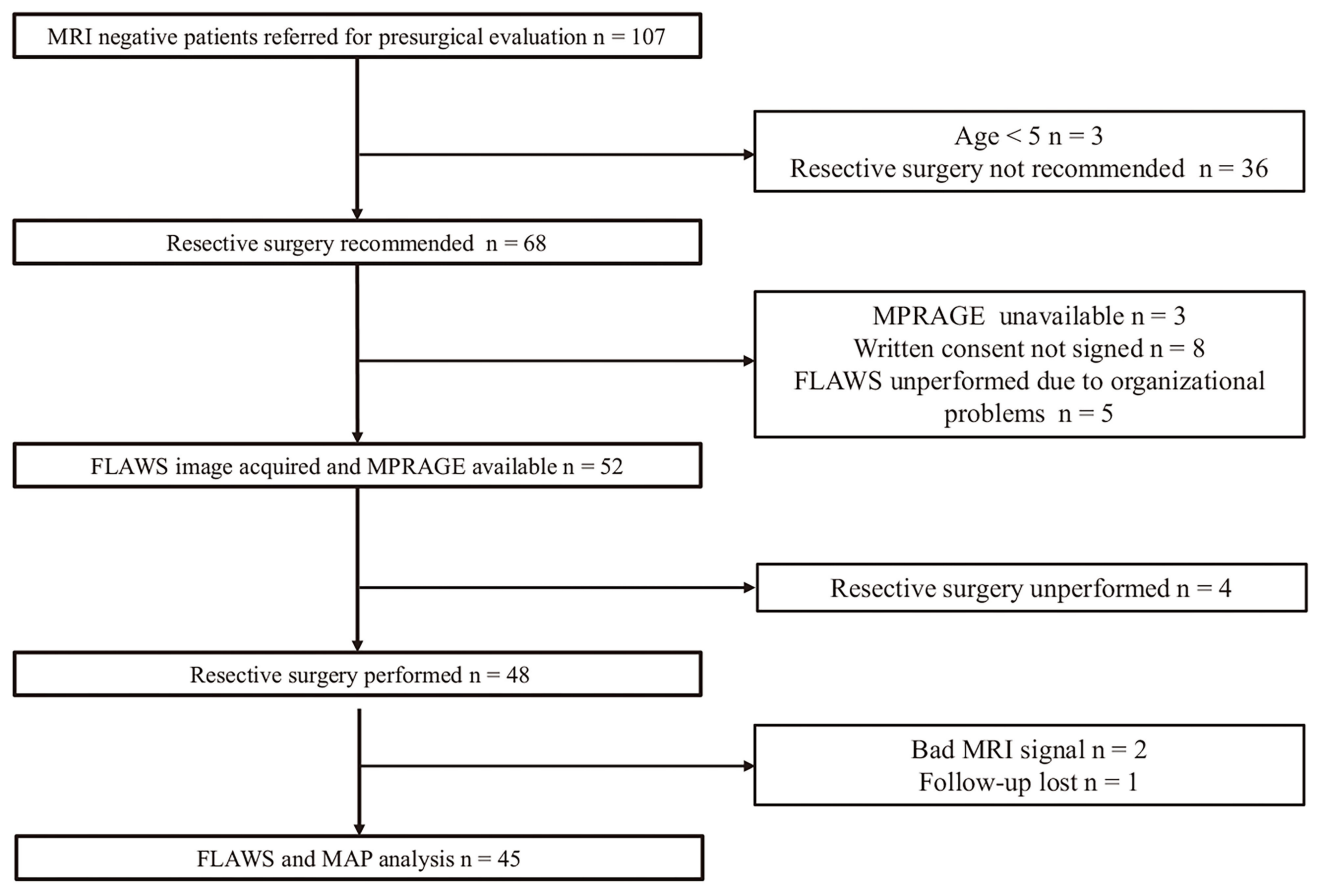
Flowchart of patient enrollment. FLAWS unperformed due to organizational problems: the time between presurgical evaluation and invasive implantation or resective surgery was too close to allow for a FLAWS scan.

A total of 107 non-lesional patients were screened; of these, three patients <5 years old and 36 patients unsuitable for resective surgery were ruled out, with resective surgical treatment recommended for 68 patients. Out of the 68 surgical candidates, three and eight patients were ruled out either because no MPRAGE was available or written consent was not obtained, respectively. Thereafter, 57 patients signed the consent form and were included in our study. FLAWS was successfully conducted in 52 patients. Four patients who did not undergo resective surgery, two patients who had poor signal on MRI, and one patient lost to follow-up were excluded; thus, a final dataset consisting of the data of 45 patients was used for analysis (Figure 1).

Detailed demographics and clinical data are included in Table 1. Thirty (66.7%) patients were seizure-free at 12 months of follow-up. Age, age group (adult or pediatric), epilepsy duration, gender, resection type (temporal, frontal, parietal, occipital, and multilobar), type of invasive exploration (SEEG, subdural, and none), and MRI subgroups (“subtly lesional” and “strictly nonlesional”) were not significantly associated with surgical outcome.

**Table 1:** Detailed demographics and clinical data of the 45 patients studied.

**Factors**	**Summary**	**Seizure-free**	**Not seizure-free**	***p*-value**
Age, years	22.7 (mean) ± 7.90 (SD), 8–42 (range), 23 (median)	23.4 ± 7.06 (SD), range = 11–42, median = 23	21.3 ± 9.48 (SD), range = 8–41, median = 18	0.41^a^
**Age group**, ***n*** **(%)**				
Age ≥ 18 years	31 (68.9)	22	9	0.49^b^
Age <18 years	14 (31.1)	8	6	
Epilepsy duration, months	12.0 (mean) ± 6.37 (SD), 2–30 (range)	12.5 ± 6.68 (SD), range = 2–30	10.9 ± 5.76 (SD), range = 2–17	0.61^c^
**Gender**, ***n*** **(%)**				
Male	25 (55.5)	17	8	1^b^
Female	20 (45.5)	13	7	
**Resection type**, ***n*** **(%)**				
Temporal	17 (37.8)	15	3	0.21^b^
Frontal	19 (42.2)	9	9	
Parietal	6 (13.3)	4	2	
Occipital	2 (4.4)	1	1	
Multilobar	1 (2.2)	1	0	
**Invasive exploration**, ***n*** **(%)**				
SEEG	36 (80)	23	13	0.61^b^
Subdural	6 (13.3)	4	2	
None	3 (6.7)	3	0	
**MRI subgroups**, ***n*** **(%)**				
Subtly lesional	10 (22.2)	6	4	0.71^b^
Strictly non-lesional	35 (77.8)	24	11	
**Pathology**, ***n*** **(%)**				
FCD I	23 (51.1)	15	8	0.22^b^
FCD II	8 (17.8)	7	1	
FCD III	1 (2.2)	1	0	
HS	1 (2.2)	1	0	
GG	2 (4.4)	2	0	
Gliosis	8 (17.8)	4	4	
Non-identifiable	2 (4.4)	0	2	
**Pathology**, ***n*** **(%)**				
FCD	32 (71.1)	23	9	0.3^b^
Others	13 (29.9)	7	6	

*Associations between parameters and seizure outcome at 12 months were tested*.

*EEG, electroencephalography; SEEG, stereoelectroencephalography; MRI, magnetic resonance imaging; HS, hippocampal sclerosis; GG, ganglioglioma; FCD, focal cortical dysplasia; SD, standard deviation*.

a *t-test*.

b *Pearson chi-square test or Fisher's exact test*.

c *Wilcoxon rank-sum test*.

Forty-two patients underwent intracranial EEG, including 36 patients undergoing SEEG implantations and six subdural EEG implantations. Out of the 42 patients explored with intracranial EEG, 27 patients (64.3%) were seizure-free. All three patients who received resective surgery without intracranial EEG were seizure-free. No significant difference in seizure-free outcome was found between patients who received intracranial EEG vs. those who did not (*p* = 0.54). For the 30 patients in the seizure-free subgroup, 27 (90%) were explored with intracranial EEG. All 15 patients in the not seizure-free subgroup underwent intracranial EEG before resective surgery. No significant difference was found regarding the intracranial EEG exploration ratio between the seizure-free and not seizure-free subgroups (*p* = 0.82).

The pathological evaluation revealed FCD I (*n* = 23, 51.1%), FCD II (*n* = 8, 17.8%), FCD III (*n* = 1, 2.2%), hippocampal sclerosis (*n* = 1, 2.2%), ganglioglioma (*n* = 2, 4.4%), gliosis (*n* = 8, 17.8%), and non-identifiable abnormalities (*n* = 2, 4.4%). The pathology classification was not associated with surgical outcome (*p* = 0.22).

### FLAWS

#### Positive Rate

**Figure 2:**
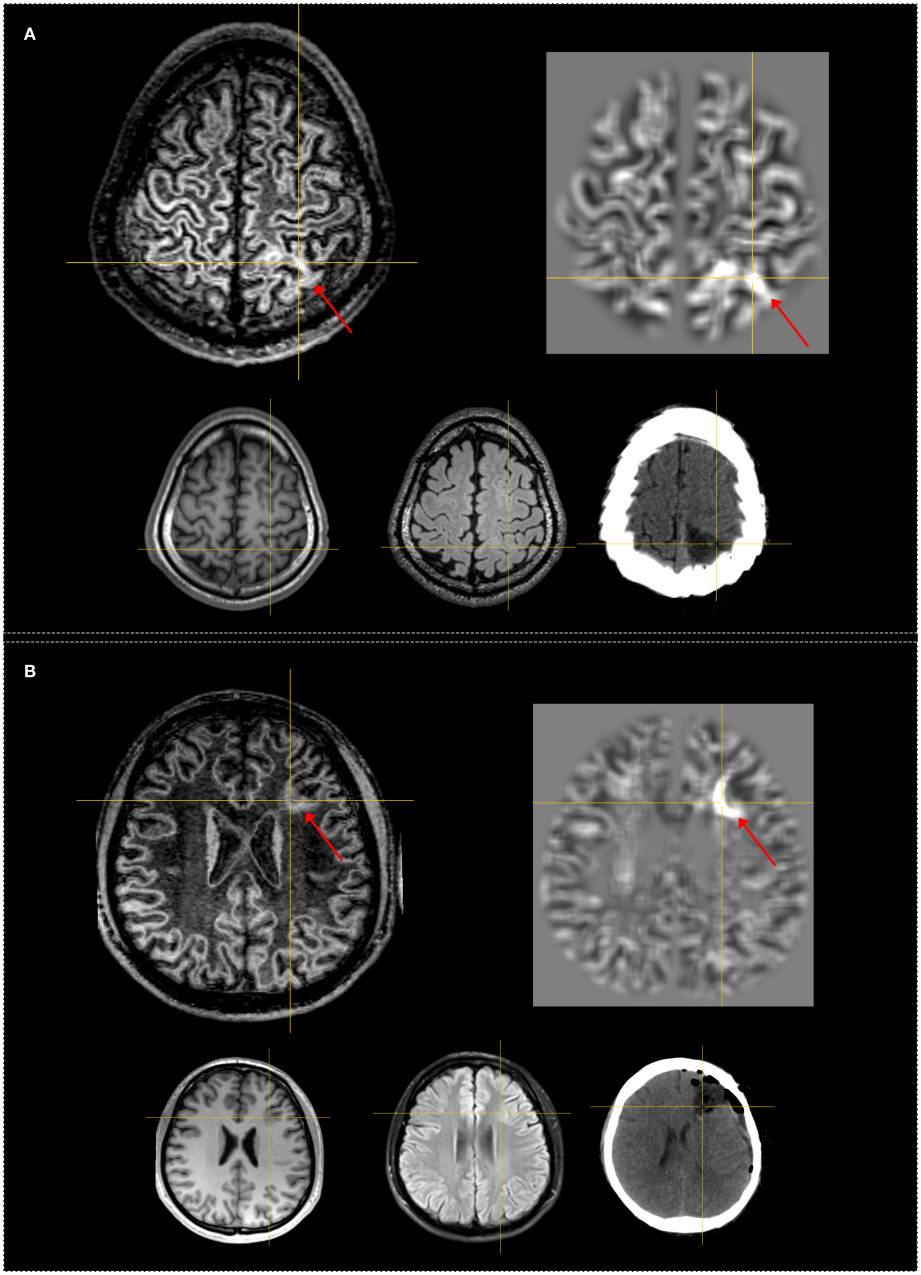
Examples of patients having FLAWS–MAP gyral+ region concordant with the surgical resection cavity. Both patients remained seizure-free at 12 months. The crosshairs indicate the same coordinate between different images. The red arrows indicate the FLAWS+ or MAP+ region. Upper, left: registered FLAWS image; right: junction map of MAP. Lower, left: T1-weighted magnetization-prepared rapid acquisition with gradient-echo (MPRAGE) image; middle: registered T2-weighted fluid-attenuated inversion recovery (FLAIR) image; right: registered postsurgical computerized tomography (CT) indicating the site and extent of resection. **(A)** belongs to the “subtly lesional” subgroup, FLAWS–MAP gyral+ region in the parietal lobe; FLAWS image shows blurring of gray matter and white matter transition as well as abnormal subcortical white matter signal. The junction file shows the suprathreshold region located in the same gyrus with FLAWS image. **(B)** belongs to the “subtly lesional” subgroup, FLAWS–MAP gyral+ region in the frontal lobe; FLAWS image shows abnormal cortical thickness, blurring of gray matter and white matter transition, and transmantle sign; the junction map shows a half ring-shaped suprathreshold region, which was located in the same sulcus with FLAWS image. Pathology: **(A)** focal cortical dysplasia (FCD) IIb; **(B)** FCD IIa.

Independent analysis revealed FLAWS+ regions in 22 (48.9%) of the 45 patients. The most frequently detected feature was “C” and the most common feature pattern was “CD.” The positive rate was 33.3% (6/18) and 57.7% (15/26) in the temporal subgroup and extratemporal subgroup, respectively. Although a higher positive rate was shown in the extratemporal subgroup, it was not statistically significant (*p* = 0.14). Figure 2 shows patients with FLAWS+ regions in different lobar regions. In the “subtly lesional” and “strictly nonlesional” subgroup, the positive rate was 70% (7/10) and 42.9% (15/35), respectively, with no significant difference (*p* = 0.17).

#### Correlation With Resection and Outcome

Correlation with resection and outcome revealed that the sensitivity, specificity, PPV, and NPV in the overall cohort were 0.43 (95% CI, 0.25–0.63), 0.87 (95% CI, 0.60–0.98), 0.87 (95% CI, 0.60–0.98), and 0.43 (95% CI, 0.25–0.63), respectively (Table 2). In 13 (86.7%) of the 15 patients, the FLAWS+ region was concordant with resection and became seizure-free; in one (14.2%) of the seven patients, the FLAWS+ region was not concordant with resection and became seizure-free. Concordance between the FLAWS+ region and resection was significantly associated with a seizure-free outcome compared with the non-concordance group (*p* = 0.002) (Table 3).

**Table 2:** Positive rate, sensitivity, specificity, PPV, and NPV in MAP, FLAWS, and FLAWS–MAP gyral concordance.

	**MAP**	**FLAWS**	**FLAWS–MAP gyral concordance**
Positive rate	64.4%	48.9%	33.3%
Sensitivity (95% CI)	0.57 (0.37–0.75)	0.43 (0.25–0.63)	0.3 (0.15–0.49)
Specificity (95% CI)	0.8 (0.52–0.96)	0.87 (0.60–0.98)	1 (0.78–1)
PPV (95% CI)	0.85 (0.62–0.97)	0.87 (0.60–0.98)	1 (0.66–1)
NPV (95% CI)	0.48 (0.28–0.69)	0.43 (0.25–0.63)	0.42 (0.26–0.59)

*CI, confidence interval; MAP, morphometric analysis program; NPV, negative predictive value; PPV, positive predictive value; FLAWS, fluid and white matter suppression*.

**Table 3:** Correlation between resection of positive regions and seizure outcomes.

	**Subgroups**	**Total**	**Seizure-free**	**Not seizure-free**	***p*-value**
MAP	Concordance	20	17	3	0.0003
	Non-concordance	9	1	8	
	Negative	16	12	4	
FLAWS	Concordance	15	13	2	0.002
	Non-concordance	7	1	6	
	Negative	23	16	7	
FLAWS MAP both gyral positive	Concordance	9	9	0	0.004
	Non-concordance	5	1	4	
	Negative	31	20	11	

*P-values were generated by Fisher's exact test. MAP, morphometric analysis program; FLAWS, fluid and white matter suppression*.

#### Pathology Findings

Surgical specimens from the 15 patients whose FLAWS+ regions were concordant with resection were pathologically diagnosed with FCD in 11 (73.3%) patients (FCD I, *n* = 6; FCD II, *n* = 5), ganglioglioma in two patients, gliosis in one patient, and non-identifiable in one patient.

### MAP

#### Positive Rate

Independent analysis revealed the presence of MAP+ regions in 29 (64.4%) of the 45 cases, of whom two (6.9%) patients had multiple MAP+ regions. In all patients with MAP+ regions, abnormalities were detected in the junction file. Two patients exhibited abnormalities in the corresponding regions in the extension file. No patient showed relevant changes in the thickness file. The positive rate was 61.1% (11/18) and 65.4% (17/26) in the temporal subgroup and extratemporal subgroup, respectively (*p* = 1). In the “subtly lesional” and “strictly nonlesional” subgroups, the positive rate was 70 and 62.8%, respectively (*p* = 1). The positive rate of MAP in patients ≥18 years old and patients <18 years old were 61.3 and 71.4%, respectively, which was not significantly different (*p* = 0.7).

#### Correlation With Resection and Outcome

Correlation with resection and outcome revealed that the sensitivity, specificity, PPV, and NPV in the overall cohort were 0.57 (95% CI, 0.37–0.75), 0.8 (95% CI, 0.52–0.96), 0.85 (95% CI, 0.62–0.97), and 0.48 (95% CI, 0.28–0.69), respectively. Seventeen of the 20 cases (85.0%), whose MAP+ region was concordant with resection, became seizure-free. One (11.1%) of the nine patients, whose MAP+ region was non-concordant with resection, became seizure-free. Concordance between resection and the MAP+ regions was significantly associated with a seizure-free outcome when compared with the non-concordance group (*p* = 0.0003) (Table 3).

#### Pathology Findings

Surgical specimens from the 20 patients whose abnormalities in MAP were concordant with resection were diagnosed with FCD in 16 (80%) patients (FCD I, *n* = 11, FCD II, *n* = 5), gliosis in two patients, hippocampal sclerosis in one patient, and ganglioglioma in one patient.

### FLAWS Correlation With MAP

#### Positive Rate

**Figure 3:**
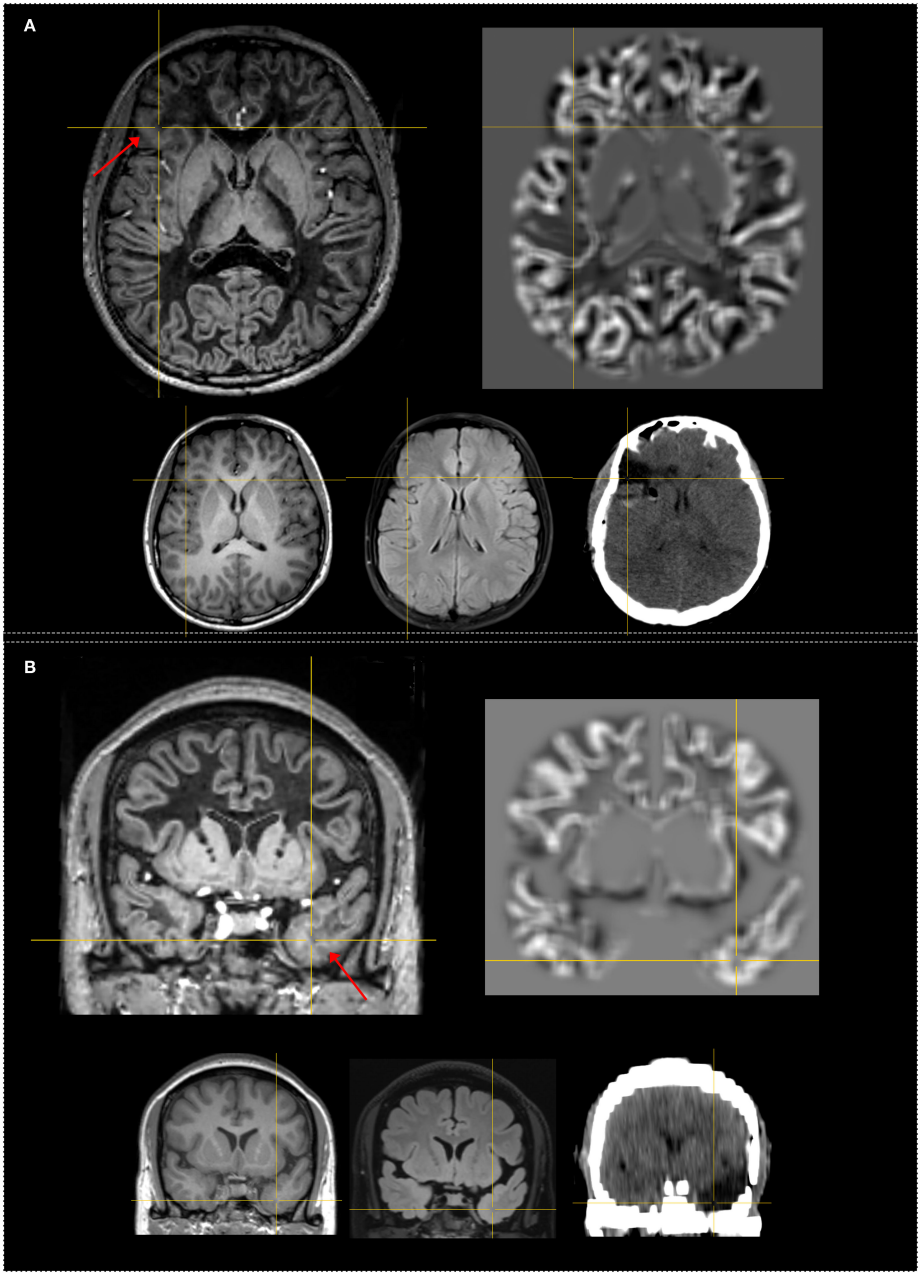
Examples of patients with only FLAWS+ region concordant with the surgical resection cavity. Both patients were seizure-free after surgery at 12 months. The crosshairs indicate the same coordinate between different images. The red arrows indicate the FLAWS+ region. Upper, left: registered FLAWS image; right: junction map of MAP. Lower, left T1-weighted magnetization-prepared rapid acquisition with gradient-echo (MPRAGE) image; middle: registered T2-weighted fluid-attenuated inversion recovery (FLAIR) image; right: registered postsurgical CT indicating the site and extent of resection. **(A)** belongs to the “strictly nonlesional” subgroup, FLAWS+ region in the frontal lobe; FLAWS image shows blurring of gray matter and white matter transition as well as abnormal cortical thickness. The junction file shows no suprathreshold region. **(B)** belongs to the “strictly nonlesional” subgroup, FLAWS+ region in the temporal lobe; FLAWS image shows blurring of gray matter and white matter transition as well as abnormal subcortical white matter signal; the junction map shows no suprathreshold region. Pathology: **(A)** FCD IIb; **(B)** gliosis.

There was no significant difference in the overall positive rate of 48.9 and 64.4% between independent FLAWS and MAP analysis (*p* = 0.29). The re-examination of abnormalities in the independent analysis with co-registered FLAWS and MAP maps revealed that 15 (33.3%) patients were positive on both FLAWS and MAP, including gyral concordance in 12 (26.6%) patients (Figure 2), lobar concordance in one (2.2%) patient, and discordance in two (4.4%) patients; FLAWS+ only (Figure 3) and MAP+ only were found in seven (15.5%) and 14 patients (31.1%), respectively; nine (20.0%) patients were negative for both FLAWS and MAP.

**Figure 4:**
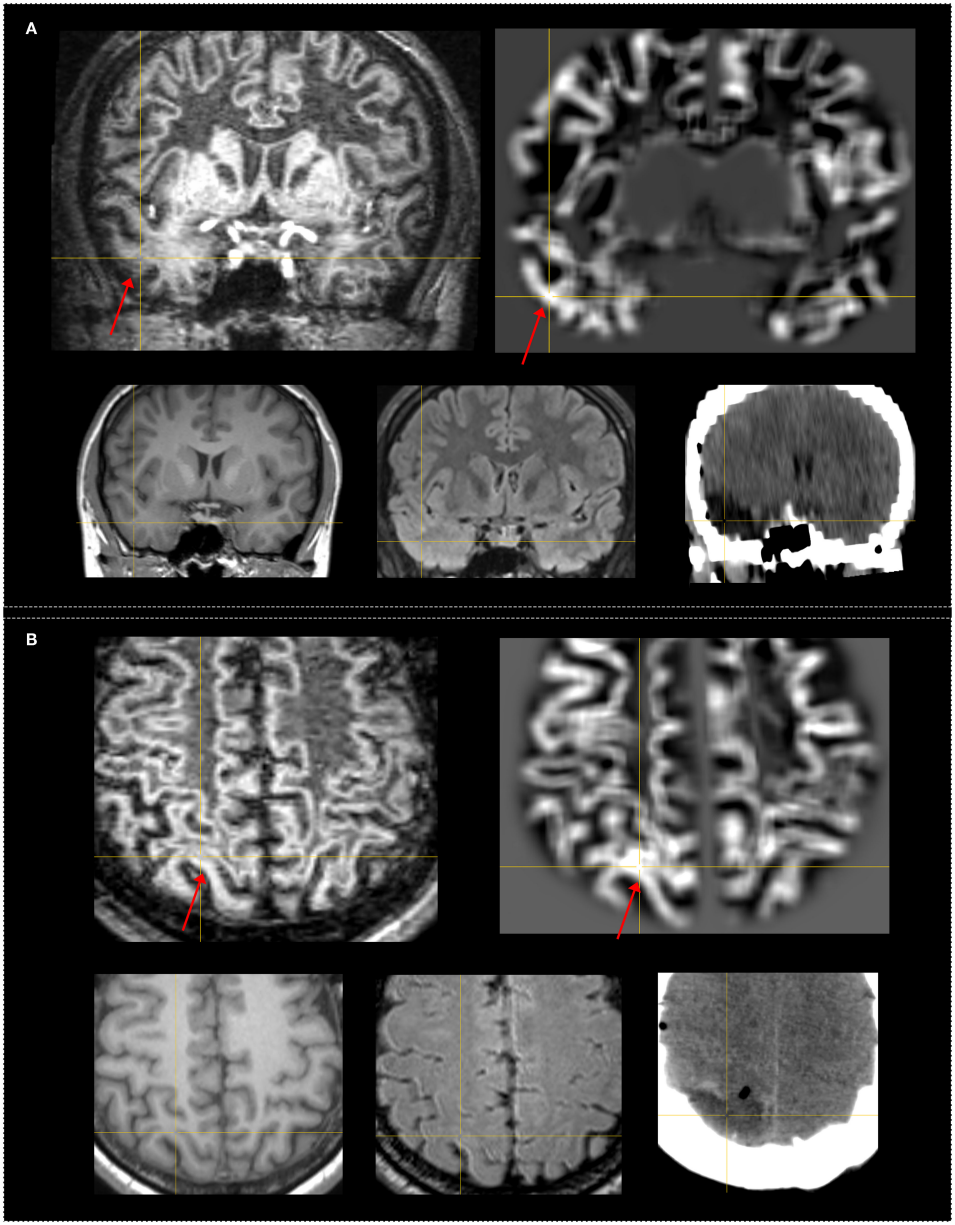
Examples of patients who had subtle FLAWS+ region revealed only under the guidance of the MAP+ region. Both patients were seizure-free after surgery. The FLAWS+ and MAP+ region was concordance with resection. The crosshairs indicate the same coordinate between different images. The red arrows indicate the FLAWS+ or MAP+ region. Upper, left: registered FLAWS image; right: junction map of MAP. Lower, left T1-weighted magnetization-prepared rapid acquisition with gradient-echo (MPRAGE) image; middle: registered T2-weighted fluid-attenuated inversion recovery (FLAIR) image; right: registered postsurgical CT indicating the site and extent of resection. **(A)** belongs to the “strictly nonlesional” subgroup, MAP guided FLAWS+ region in the temporal lobe; the junction file shows the suprathreshold region in the temporal lobe; FLAWS image shows subtle blurring of gray matter and white matter transition as well as abnormal subcortical white matter intensity. **(B)** belongs to the “subtly lesional” subgroup, MAP guided FLAWS+ region in the parietal lobe; the junction file shows the suprathreshold region in the parietal lobe; FLAWS image shows very subtle blurring of gray matter and white matter transition. Pathology: **(A)** FCD Ib; **(B)** FCD Ia.

Moreover, two patients considered negative in independent FLAWS analysis were found to have an abnormality on FLAWS in the corresponding regions guided by MAP+ regions. The gyral FLAWS–MAP concordance positive rate was consequently 31.1% (14 out of 45 patients). In both patients, the abnormality of GM–WM junction blurring was very subtle and located in a very small region. Therefore, these features escaped the initial visual FLAWS inspection (Figure 4).

#### Correlation With Resection and Outcome

Correlation with resection and outcome showed that FLAWS–MAP gyral concordance-positive (FLAWS–MAP gyral+) in the overall cohort had a sensitivity, specificity, PPV, and NPV of 0.3 (95% CI, 0.15–0.49), 1 (95% CI, 0.78–1), 1 (95% CI, 0.66–1), and 0.42 (95% CI, 0.26–0.59), respectively. Nine of the nine patients (100%) whose FLAWS–MAP gyral+ region was concordant with resection became seizure-free, and one of the five patients (20%) whose FLAWS–MAP gyral+ region was not concordant with resection became seizure-free. Concordance between resection and the FLAWS–MAP gyral+ regions was significantly associated with a seizure-free outcome, compared with the non-concordance group (*p* = 0.004) (Table 3).

#### Pathology Findings

Surgical specimens from the nine patients whose abnormalities in FLAWS–MAP gyral+ region were concordant with resection were diagnosed with FCD in eight (88.9%) patients (FCD I, *n* = 4; FCD II, *n* = 4) and with ganglioglioma in one patient.

## Discussion

In this study, we evaluated the potential diagnostic value of a novel MRI sequence (FLAWS) in comparison with a well-developed postprocessing method (MAP) among patients with MRI-negative epilepsy. Our main findings were as follows: (1) FLAWS could detect subtle abnormalities with high specificity (0.87) and low sensitivity (0.43), (2) MAP could detect subtle abnormalities with a higher sensitivity (0.57) and a similar specificity (0.80) as those of FLAWS, and (3) a complementary value from FLAWS existed in MAP-negative patients.

### FLAWS

#### Positive Rate of FLAWS in Epilepsy

The application of FLAWS in epilepsy has been rarely reported. We have previously reported that the sensitivity and specificity of FLAWS in patients with conventional MRI-positive and MRI-negative FCD were 71.9 and 71.1%, respectively (8). By comparison, the relatively lower sensitivity in our study was due to the conventional MRI-negative nature of the included patients. The usage of FLAWS to detect cases of suspected FCD and heterotopic neurons outside the cortex was also suggested in a review (31). To date, no other reports on FLAWS in epilepsy are available.

#### FLAWS in MRI-Negative Epilepsy

In patients with normal appearance on conventional MRI, lesions are usually subtle and difficult to detect. We found that the most frequent detected single feature was “blurred junction of GM–WM” and the most common feature combination pattern was “blurred junction of GM–WM with abnormal signal intensity of subcortical WM.” Owing to the highly convoluted nature of the cortex, a proportion of certain images may contain a mixture of several tissues, such as WM, CSF, and GM (32). CSF and WM suppression allows GM visualization with minimal WM and CSF influence. Such a strategy could highlight abnormalities, especially abnormalities in the GM–WM transition, which was the likely mechanism underlying the discovery of subtle lesions using FLAWS in MRI-negative patients.

#### FLAWS Compared With Double Inversion Recovery

The strategy of suppressing the WM and CSF signals was also employed in the double inversion recovery turbo spin-echo sequence previously developed (33). Double inversion recovery (DIR) images can depict cortical tubers (34), demonstrate WM abnormal signals in temporal lobe epilepsy (35), and have achieved a sensitivity and specificity of 0.88 and 0.88, respectively, in a cohort composed of patients with MRI-negative and MRI-positive FCD (36). In patients with negative conventional MRI findings, visual inspection of DIR could also be used to detect lesions concordant with EEG (37), and a positive rate of 45.5% was reported using voxel-by-voxel statistical analysis with DIR (32). Similar with our findings, qualitative analyses found that DIR was remarkably able to reveal “blurring and subcortical WM abnormalities” (38).

A direct comparative study between DIR and FLAWS in epilepsy was unavailable. However, one technique study reported that FLAWS was advantageous for having a single inversion pulse preparation and small flip angle gradient-recalled echo readout compared with DIR sequence, which has two inversion pulse preparation and a large flip angle turbo spin-echo readout. Ultra-high field 7 T MRI scans in healthy subjects revealed that FLAWS exhibited more homogeneous WM suppression and better GM visualization than DIR (39). Further studies are needed to assess the value of these two sequences in epilepsy.

### MAP in MRI-Negative Epilepsy

In this study, MAP revealed subtle abnormalities in 64.4% of patients, a finding generally consistent with previous literature (17, 28, 40, 41). Detection rates of 45% for MRI-negative patients in insular epilepsy (42), 24.2% in extratemporal lobe epilepsy (43), 85% in FCD type II (24), and 43% in a large epilepsy cohort (17) have been reported using MAP. The slight variation of MAP detection rate among these studies was due to the difference in the definition of “MRI-negative” and the composition of patients included.

MAP generates junction, extension, and thickness maps after normalization, segmentation, the convolution of individual patient's image, and the comparison of individual patient's image with a database of normal controls. Through these steps, the output maps indicate blurring or aberrant GM beyond the cortical ribbon with hyperintensity (14, 15) and have the advantage of their high sensitivity because of the ability of highlighting subtle changes and which can easily escape visual inspection.

### FLAWS Correlation With MAP

#### Superiority of MAP to Current FLAWS Analysis

In our study, MAP alone had a higher sensitivity than FLAWS alone, and the exclusive yield was higher with MAP than in FLAWS (14 in MAP, seven in FLAWS). In addition, two additional patients, negative in the independent visual FLAWS analysis, became positive under the MAP guidance. The abnormalities on the FLAWS images of these two patients were located in a very limited region and consisted of a few connected gyri (Figure 4), which led to the miss in the independent visual inspection. It is not surprising that independent MAP analysis showed these advantages over independent FLAWS analysis, considering the different nature of these two techniques. MAP facilitates the detection of subtle changes by statistical comparison, whereas the current visual FLAWS inspection in this study was manual in nature. Thus, the advantage of MAP to FLAWS in this study partly reflected the superiority of algorithm-assisted morphological analysis over manual visual identification. Besides, an extra scan is required for FLAWS. However, MAP can be performed using MPRAGE images without additional cost or risk, making it more cost-effective.

#### The Potential Value of FLAWS in MAP-Negative Patients

In addition to the superiority of MAP, there are two other crucial findings from our study. The first was the complementary value of FLAWS for MAP analysis. Seven patients had abnormal findings in FLAWS analysis only, which indicated the unique value of the FLAWS sequence. In the comparison of the FLAWS image with the MPRAGE one, as shown in Figure 3, WM suppression highlighted the blurring of the GM–WM junction and uncovered a hyperintensity in the surrounding subcortical WM. Thus, the gain of FLAWS over MAP in our study resulted from the gain of WM suppression in individual FLAWS over MPRAGE images. Subtle lesions only or mainly exhibiting on the T2 signal were also undetected by MAP in subtly lesional MRI-negative patients because of the chosen MPRAGE input in MAP analysis (17). These findings suggested the usefulness of a voxel-based morphometric analysis using FLAWS images in the future.

#### Using FLAWS With Current MAP in Practice

Another important finding was the revelation of a high gyral FLAWS–MAP concordance. Despite the difference in methodology between these two techniques, 31.1% of patients showed gyral FLAWS–MAP concordance. Considering the surgical outcome and pathological findings, we believe that common structural changes rather than coincidences are underlying these co-detected abnormalities.

Abnormalities on MAP maps represent the deviation from a normal distribution; therefore, suprathreshold regions may be caused by registration errors, imaging artifacts, or normal variants. Assessment of subtle but visually identifiable changes using T1, T2, and FLAIR images is the usual method for validating MAP results (17, 41, 44). Using positron emission tomography or magnetoencephalography to confine the MAP findings by adding functional or electrical information is another way (18, 28). As a structural image that provides new information, even when voxel-based morphometric analysis based on FLAWS is unavailable, there are still practical implications using FLAWS with the current MAP. However, the low sensitivity and high PPV of FLAWS–MAP gyral+ suggested that, when encountered, the finding would be a vital preimplantation hypothesis and subsequently helpful in optimizing the stereoelectroencephalography implantation and surgical strategy for the challenging cohort of MRI-negative patients.

In general, patients with negative MRI results have poorer postoperative outcomes and may not be referred for surgical treatment. Accurate detection and delineation of lesions cannot be overstated. Rapidly evolving postprocessing methods using currently available mature MRI sequences such as MPRAGE, T2, and FLAIR have augmented and improved the detection of subtle lesions, including FCD and other pathological diseases. However, the findings of complementary yields by visual FLAWS inspection to MPRAGE-based MAP analysis and the high PPV of FLAWS–MAP gyral+ abnormalities observed in our study suggest novel MRI sequences still worth exploring.

### Limitation

Our study has several limitations. Our study is limited in that only postoperative CT was available. Further studies with postoperative MRI are needed to verify our findings. A voxel-based morphometric statistical analysis using FLAWS images to compute the deviation of individual patient's image to normal distribution was not performed, because a large number of age-matched healthy volunteers would be needed. A comparison of FLAWS to T2 or FLAIR-based MAP analysis was also not performed in our study, because only high-resolution MPRAGE images were available in our hospital. Further studies are warranted to prompt more comprehensive results. Furthermore, normal pediatric databases were not available in this study. Although no significant difference was found between the age ≥18 and age <18 groups in the present study, age-specific normal databases for voxel-based morphometric analysis are advisable.

## Conclusions

FLAWS, as a new tool, showed a promising value for identifying subtle epileptogenic lesions. It can also be used as a complement to current MAP in patients with MRI-negative epilepsy.

## Data Availability Statement

The raw data supporting the conclusions of this article will be made available by the authors, without undue reservation.

## Ethics Statement

The studies involving human participants were reviewed and approved by Ethics Committee of Xuanwu Hospita. Written informed consent to participate in this study was provided by the participants' legal guardian/next of kin.

## Author Contributions

KS analyzed the clinical data, reviewed the FLAWS and MAP with postsurgical CT image, and wrote the manuscript. TY, LQ, and DN performed the surgical treatment. DY reviewed the MAP maps and revised the manuscript. ZR collected the FLAWS data. XW reviewed the MAP maps. YZ revised the manuscript and reviewed the FLAWS image. XC collected the FLAWS data, reviewed the FLAWS image, and revised the manuscript. JX revised the manuscript. NC reviewed the FLAWS image. RG and YL reviewed the literature. KY participated in the data statistical analysis. TK designed the FLAWS sequence. GZ conceived the study, supervised the data analysis, and revised the manuscript. All authors contributed to the article and approved the submitted version.

## Conflict of Interest

TK is a full employee of Siemens Healthcare AG Switzerland. The remaining authors declare that the research was conducted in the absence of any commercial or financial relationships that could be construed as a potential conflict of interest.

## Abbreviations

CSF: cerebral spinal fluid
CT: computed tomography
DIR: double inversion recovery
EEG: electroencephalogram
FCD: focal cortical dysplasia
FLAIR: fluid-attenuated inversion recovery
FLAWS: fluid and white matter suppression
GM: gray matter
MAP: morphometric analysis program; multidisciplinary presurgical evaluation conference
MPRAGE: magnetization-prepared rapid acquisition with gradient echo
MRI: magnetic resonance imaging
PPV: positive predictive value
WM: white matter.
