# Supplementary material for: Fluid and White Matter Suppression Imaging and Voxel-Based Morphometric Analysis in Conventional Magnetic Resonance Imaging-Negative Epilepsy
Source: Front Neurol. 2021 Apr 29;12:651592. doi: 10.3389/fneur.2021.651592 (PMC8116947; doi:10.3389/fneur.2021.651592)
Supplement: Supplementary file 1 [file Data_Sheet_1.PDF]

Figure S1. Analysis procedure. There were three steps in analysis. Step one: independent original FLAWS or MAP analysis. Step two: co-registrated FLAWS or MAP overlaid with co-registrated postoperative CT. Step three: a joint re-review of co-registered FLAWS with MAP.

Figure S2. Overview of the image processing steps required to compare the resection cavity and detected abnormalities.

A. Registration. FLAWS and postoperative CT were registered onto the preoperative MPRAGE, which made all the images in the same coordinate system.

B. Based on postoperative CT, the resection cavity could be delineated using the segmentation module in 3D slicer to visualize surgical margins.

C. Assess the relationship between the resection cavity and FLAWS or MAP abnormalities. After visualizing the surgical margin on FLAWS and MAP maps, the distance between the border of the abnormalities and the resection margin can be measured with ruler in 3D slicer.
