# Supplementary figures and images for: Fluid and White Matter Suppression Imaging and Voxel-Based Morphometric Analysis in Conventional Magnetic Resonance Imaging-Negative Epilepsy
Source: Front Neurol. 2021 Apr 29;12:651592. doi: 10.3389/fneur.2021.651592 (PMC8116947; doi:10.3389/fneur.2021.651592)

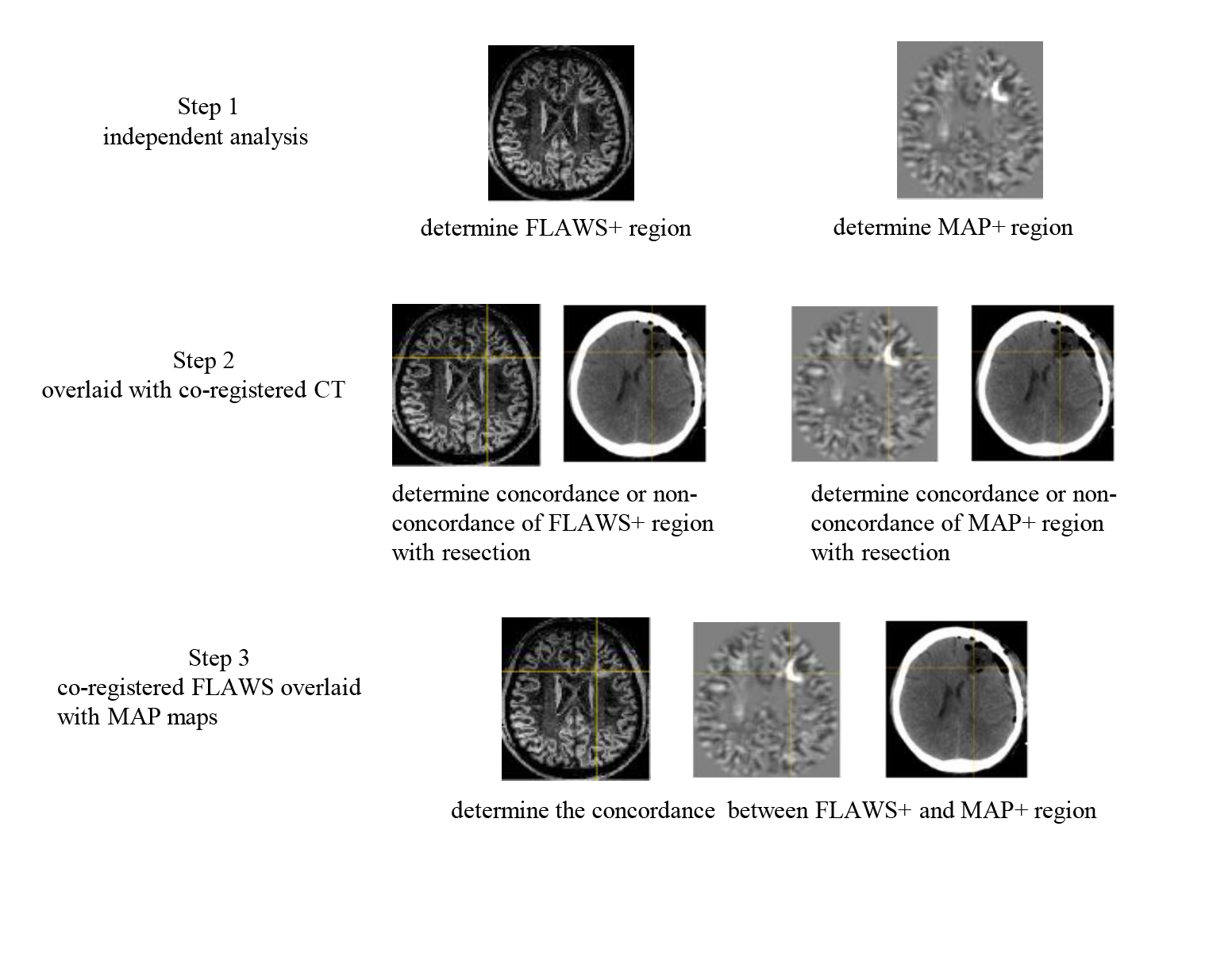

Supplement: Supplementary file 2 [file Image_1.TIF]

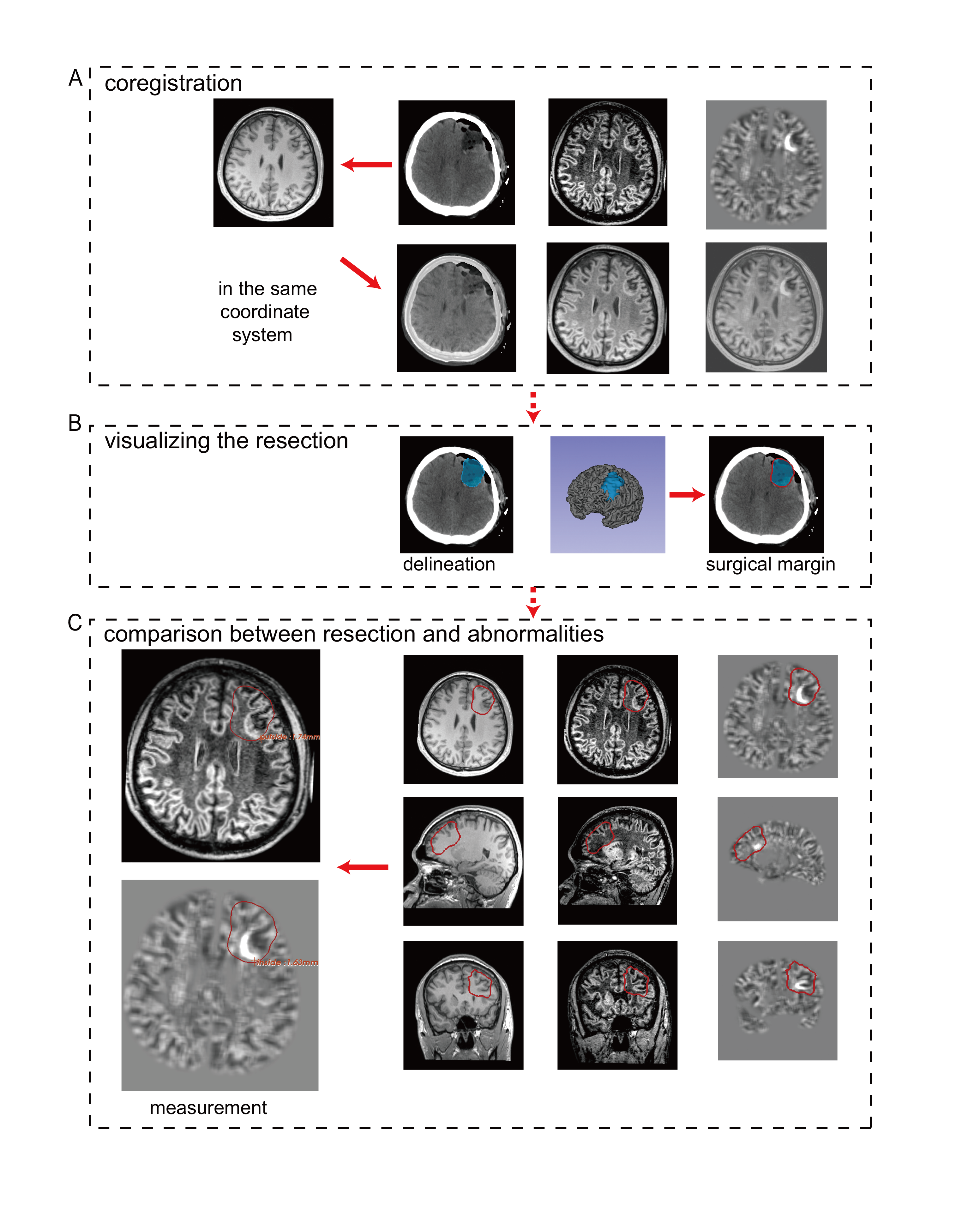

Supplement: Supplementary file 3 [file Image_2.TIF]
